# *BAP1* methylation: a prognostic marker of uveal melanoma metastasis

**DOI:** 10.1038/s41698-021-00226-8

**Published:** 2021-09-30

**Authors:** Mathieu F. Bakhoum, Ellis J. Curtis, Michael H. Goldbaum, Paul S. Mischel

**Affiliations:** 1 Department of Ophthalmology and Visual Science, Yale University School of Medicine, New Haven, CT USA; 2 Department of Ophthalmology, University of California San Diego, La Jolla, CA USA; 3 School of Medicine, University of California San Diego, La Jolla, CA USA; 4 Department of Pathology, Stanford University School of Medicine, Stanford, CA USA; 5 ChEM-H, Stanford University, Stanford, CA USA

**Keywords:** Prognostic markers, Cancer epigenetics

## Abstract

Uveal melanoma, the most common intraocular primary cancer in adults, is characterized by striking variability in metastatic tendencies*. BAP1* deletion in the primary tumor is associated with uveal melanoma metastasis, but it cannot always be resolved by bulk DNA sequencing of heterogeneous tumors. Here, we show that assessment of *BAP1* methylation is an accurate and readily clinically actionable assay to accurately identify high-risk uveal melanoma patients.

## Introduction

Uveal melanoma (UM), the most common intraocular primary cancer in adults, is characterized by striking variability in metastatic tendencies^1^. Tests that assess the metastatic proclivity of the primary tumor provide individualized prognostication and help guide metastatic surveillance. UM tumors with high metastatic tendencies differ from their more indolent counterparts in several ways^1,2^. They often harbor mutations in the *BAP1* gene, lack one copy of chromosome 3 and have a characteristic transcriptome and methylome^3–9^. In current clinical practice, prognostication methods rely on assessing a single or a combination of these features. RNA-based tests include a clinically validated 12-gene signature representative of the wide transcriptional changes that distinguish the two prognostic groups; low-risk (Gene expression profile 1, GEP1) and high-risk (Gene expression profile 2, GEP2)^9^. DNA-based tests can detect genomic alterations in the *BAP1* gene and identify chromosome copy number alterations (chromosomes 3, 6, and 8). Immunohistochemical assessment of BAP1 staining or cellular morphology can also be employed to estimate the risk of metastasis^10^.

UM tumors are heterogeneous; cells within a single tumor can have different prognostic features including variable expression levels of BAP1^10–12^. Hence, prognostic tests that classify UM into two major groups lack the resolution to assess the diversity of an inherently heterogeneous tumor. In fact, detailed analysis of the TCGA UM cohort (integrative analysis of UM transcriptomes, methylomes and genomic copy number data, *n* = 80 patients) has revealed the existence of four molecularly distinct biological and prognostic subsets of UM^3^.

Conversely, methods that can accurately assess cellular heterogeneity, i.e. BAP1 staining or chromosome 3 fluorescent in situ hybridization (FISH), require specialized technical skills. An affordable and reproducible bulk test that yet has the ability to assess the tumor’s heterogeneity, would offer an attractive alternative.

**Fig. 1:**
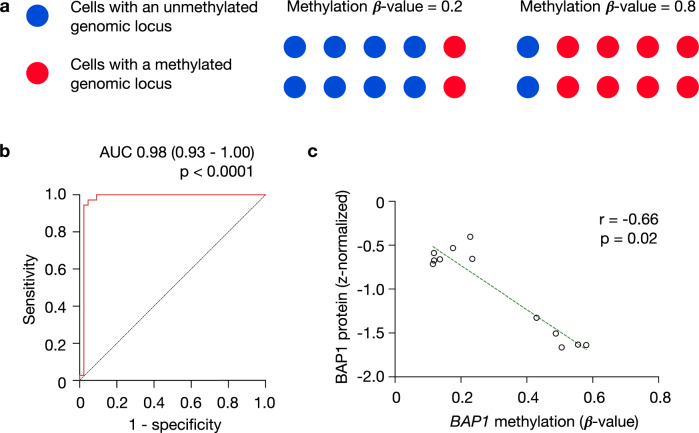
BAP1 methylation—a surrogate for BAP1 genomic copy number and transcript levels. **a** β-methylation values are indicative of the fraction of cells that are methylated within the specimen at that specific locus. **b** ROC curve for the association between *BAP1* methylation (derived from Infinium HumanMethylation450K BeadChip arrays) and *BAP1* genomic copy loss (data accessed from cbioportal^15,16^). The reference line for random prediction is shown (dotted). AUC, area under the ROC curve, with 95% confidence intervals. **c** BAP1 protein levels plotted as a function of *BAP1* β-methylation values among 12 primary UM tumors from the TCGA-UM cohorts, with reported Spearman’s rank correlation coefficient. Dotted line represents regression line.

*BAP1* is subject to epigenetic modifications, and its hypermethylation at chromosome 3: 52,408,017 (GRCh38) is inversely correlated with *BAP1* mRNA expression and is enriched in GEP2 UMs^3,13^ (Supplementary Table 1). DNA-methylation of a given genomic locus within an individual cell is a binary state. Hence, methylation levels (β-values) obtained from a biological specimen indicate the fraction of cells that are methylated within the specimen at that specific locus (Fig. 1a). We then asked whether methylation levels of *BAP1* could be employed as a surrogate of the preponderance of tumor subclones with high metastatic potential.

First, we examined the relationship between *BAP1* methylation values and *BAP1* genomic copy loss as an outcome, using the receiver-operating-characteristic (ROC) curve. There was a significant association between *BAP1* genomic copy loss and its methylation values, with an area under the ROC curve of 0.98 (95% CI, 0.93 to 1.00; *p* < 0.0001) **(**Fig. 1b**)**. This suggests that *BAP1* methylation can be used as a surrogate of its genomic copy loss, which in turn is strongly associated with UM metastasis^3^.

**Fig. 2:**
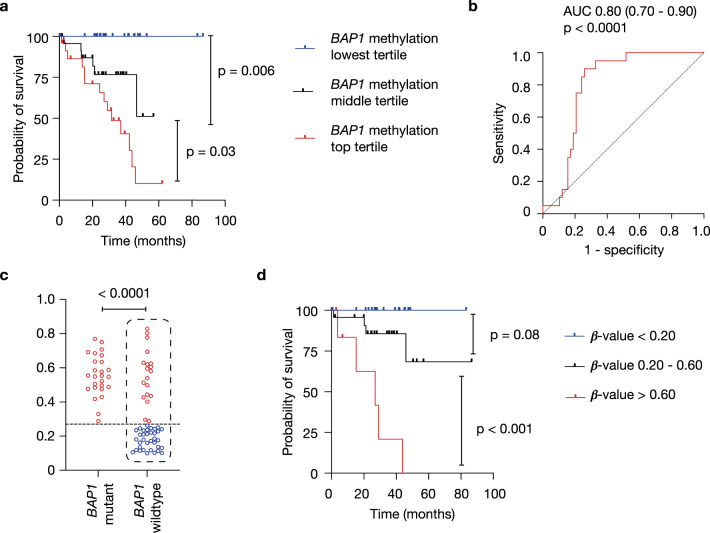
High BAP1 methylation levels correlate with worse survival. **a** Probability of survival of (*n* = 80) TCGA-UM subjects with primary tumors stratified by *BAP1* β-methylation values into three tertiles. Statistical significance tested using two-sided log-rank test. **b** ROC curve for the association between *BAP1* methylation and death. The reference line for random prediction is shown (dotted). AUC, area under the ROC curve, with 95% confidence intervals. **c** *BAP1* methylation levels in *BAP1*-mutant (*n* = 26) and BAP1-wildtype (*n* = 54) UM tumors, as identified by whole-exome sequencing (data accessed from cbioportal^14,15^). Statistical significance tested using two-sided Student’s *t* test. A dotted horizontal line at *BAP1* β-methylation value of 0.27 is shown. **d** Probability of survival of TCGA-UM subjects with *BAP1*-wildtype tumors (shown in dotted box in C, *n* = 54) with primary tumors stratified by their *BAP1* β-methylation values lower (*n* = 35) and higher (*n* = 19) than 0.27, shown in blue and red, respectively. Statistical significance tested using two-sided log-rank test.

BAP1 immunohistochemical staining of UMs demonstrates that the fraction of BAP1-positive cells offers useful prognostic information independent of other predictors^10^, further underscoring the importance of assessing the tumor’s heterogeneity in prognostication. We found that *BAP1* methylation levels are inversely correlated with BAP1 protein levels obtained from twelve tumors in The Cancer Genome Atlas (TCGA)-UM cohort (*r* = −0.66, *p* = 0.02, Fig. 1c), suggesting that *BAP1* methylation is indicative of low BAP1 expression levels. We then sought to test whether *BAP1* methylation values, surrogates of the preponderance of aggressive tumor subclones, could also offer prognostic information. We subdivided subjects from the TCGA-UM cohort (*n* = 80) into three different tertiles based on the primary tumor’s *BAP1* methylation values. Indeed, we found that the higher the percentage of *BAP1* methylation, the worse the prognosis (*p* < 0.0001, Fig. 2a).

Mutations in the *BAP1* gene confer poor prognosis. However, it can be difficult to detect intronic *BAP1* mutations or deletions using whole-exome sequencing. We then thought to determine whether *BAP1* methylation levels could offer additional prognostic information when no *BAP1* mutations are detected using whole-exome sequencing. We analyzed the relationship between *BAP1* methylation β-values and death an outcome, using the ROC curve. There was a significant association between *BAP1* methylation values and death, with an area under the ROC curve of 0.80 (0.70 to 0.90; *p* < 0.0001) (Fig. 2b). The optimal *BAP1* methylation β-value to predict survival in UM subjects was 0.27, as determined using the Youden index method^14^. While *BAP1*-mutant UMs had higher *BAP1* methylation β-values than *BAP1*-wildtype tumors, a subset of *BAP1*-wildtype UMs had *BAP1* methylation β-values higher than 0.27 (Fig. 2c). We then sought to determine whether stratifying tumors based on their *BAP1* methylation levels, using different cut off values, <0.20, 20 to 60 and >60, could offer additional prognostic information in TCGA-UM tumors where no *BAP1* mutations were detected using whole-exome sequencing. Indeed, we found that the higher the percentage of *BAP1* methylation, the worse the prognosis, even among tumors with no detectable *BAP1* mutations (*p* < 0.0001, Fig. 2c).

UMs with high metastatic tendencies have a characteristic transcriptome and methylome^3–9^. We then sought to determine whether utilizing a genome-wide methylation panel would provide superior prognostic information as compared to relying on *BAP1* single-locus methylation alone. We identified the top 1% hypermethylated CpG loci in monosomy vs disomy 3 tumors (*n* = 4,856). Interestingly, stratifying TCGA-UM tumors (*n* = 80) based on the median methylation value of *BAP1* alone, was associated with a higher hazard ratio (HR) of survival compared to relying on the larger methylation panel, HR 27.4 (95% CI, 11.2–66.8) vs 13.0 (5.3–31.7) (Supplementary Fig. 1).

In summary, our analysis suggests that *BAP1* methylation at a single genomic locus strongly correlates with *BAP1* mutations, *BAP1* genomic copy loss and its protein levels. Importantly, it provides useful prognostic information when used as a stand-alone test, even in tumors where no *BAP1* mutations were detected using whole-exome sequencing. While monosomy 3 tumors have a distinctive methylome, incorporating the methylation levels of additional genome-wide loci in the test did not lead to additional prognostic value. There are several aspects that poise this method as a prognostication test with clinical utility. It provides individualized and accurate prognostication based on the extent of the specified locus methylation. It relies on bulk tumor analysis while also acting as a surrogate of the tumor’s heterogeneity, as the methylation *β*-values obtained from bulk tumor analysis represents the fraction of cells that are methylated at that locus. Cost and technical skills are major limitations of current tests which have limited their widespread use, whereas assessing methylation status at a single genomic locus is reproducible and much more affordable. Finally, as a DNA-based test, handling of specimens is less technically cumbersome than RNA-based tests. The test can be applied on frozen or formalin-fixed specimens, and methylation status can be obtained from targeted amplicon sequencing after bi-sulfite conversion, or from DNA-methylation arrays. Fresh specimens can also be diluted and do not need to be shipped on dry ice. In summary, the data presented here nominate *BAP1* methylation as a streamlined, highly informative, cost-effective, and readily actionable test that can be performed on bulk tumor samples and that should be prospectively evaluated for its value as a stand-alone prognostic test to identify high-risk UM patients.

## Methods

### Statistical analysis

Statistical analyses were performed using R (Vienna, Austria) and GraphPad Prism version 9.2.0 (San Diego, California USA). The associations between *BAP1* methylation (derived from Infinium HumanMethylation450K BeadChip arrays) and *BAP1* genomic copy loss, as well as *BAP1* methylation and death were analyzed with the receiver-operating-characteristic (ROC) curves. Area under the ROC curve (AUC) was reported with 95% confidence intervals using GraphPad Prism version 9.2.0 (San Diego, California USA). The association between BAP1 protein levels and *BAP1* β-methylation values was tested using Spearman’s rank correlation. When comparing survival outcomes between groups we utilized two-sided log-rank test. The hazard ratio was reported with 95% confidence intervals. When comparing variables between two groups we utilized two-sided Student’s *t* test.

### Ethics statement

The study adhered to the tenets of the Declaration of Helsinki and was conducted in accordance with the regulations of the Health Insurance Portability and Accountability Act. Internal Review Board (IRB) approval was obtained from the University of California San Diego Health System.

### Reporting summary

Further information on research design is available in the Nature Research Reporting Summary linked to this article.

## Supplementary information

Supplementary Information

Reporting Summary

## Data Availability

All genomic and clinical data presented here is from the TCGA-UM cohort which is accessible through cbioportal.org^15,16^, [http://www.cbioportal.org/study/summary?id=uvm_tcga] and the Genomic Data Commons Data Portal portal.gdc.cancer.gov. Source data used are available from the corresponding author upon reasonable request.

## References

[1] Jager MJ (2020). Uveal melanoma. Nat. Rev. Dis. Prim..

[2] Bakhoum, M. F. & Esmaeli, B. Molecular characteristics of uveal melanoma: insights from the Cancer Genome Atlas (TCGA) Project. *Cancers (Basel)* **11**, 10.3390/cancers11081061 (2019). 10.3390/cancers11081061 PMC6721321 31357599

[3] Robertson AG (2017). Integrative analysis identifies four molecular and clinical subsets in uveal melanoma. Cancer Cell.

[4] Harbour JW (2010). Frequent mutation of BAP1 in metastasizing uveal melanomas. Science.

[5] Ferrier, S. T. & Burnier, J. V. Novel methylation patterns predict outcome in uveal melanoma. *Life (Basel)* **10**, 10.3390/life10100248 (2020). 10.3390/life10100248 PMC7589184 33092094

[6] Prescher G (1996). Prognostic implications of monosomy 3 in uveal melanoma. Lancet.

[7] Horsman DE, Sroka H, Rootman J, White VA (1990). Monosomy 3 and isochromosome 8q in a uveal melanoma. Cancer Genet. Cytogenet..

[8] Tschentscher F (2003). Tumor classification based on gene expression profiling shows that uveal melanomas with and without monosomy 3 represent two distinct entities. Cancer Res..

[9] Onken MD, Worley LA, Ehlers JP, Harbour JW (2004). Gene expression profiling in uveal melanoma reveals two molecular classes and predicts metastatic death. Cancer Res..

[10] Szalai E, Wells JR, Ward L, Grossniklaus HE (2018). Uveal melanoma nuclear BRCA1-associated protein-1 immunoreactivity is an indicator of metastasis. Ophthalmology.

[11] Stalhammar G, See TRO, Phillips SS, Grossniklaus HE (2019). Density of PAS positive patterns in uveal melanoma: Correlation with vasculogenic mimicry, gene expression class, BAP-1 expression, macrophage infiltration, and risk for metastasis. Mol. Vis..

[12] Bakhoum, M. F. et al. Loss of polycomb repressive complex 1 activity and chromosomalinstability drive uvealmelanoma progression. *Nat. Commun.* **12**, 10.1038/s41467-021-25529-z (2021).. 10.1038/s41467-021-25529-z PMC8438051 34518527

[13] Field MG (2019). BAP1 loss is associated with DNA methylomic repatterning in highly aggressive class 2 uveal melanomas. Clin. Cancer Res..

[14] Youden WJ (1950). Index for rating diagnostic tests. Cancer.

[15] Gao J (2013). Integrative analysis of complex cancer genomics and clinical profiles using the cBioPortal. Sci. Signal.

[16] Cerami E (2012). The cBio cancer genomics portal: an open platform for exploring multidimensional cancer genomics data. Cancer Discov..

