# Supplementary material for: BAP1 methylation: a prognostic marker of uveal melanoma metastasis
Source: NPJ Precis Oncol. 2021 Sep 30;5:89. doi: 10.1038/s41698-021-00226-8 (PMC8484429; doi:10.1038/s41698-021-00226-8)
Supplement: Supplementary file 1 — Supplementary Information [file 41698_2021_226_MOESM1_ESM.pdf]

Supplementary Information

Title: *BAP1* methylation: a prognostic marker of uveal melanoma metastasis

Supplementary Table 1. The relationship between *BAP1* mRNA levels and β-methylation values at different CpG loci located within the *BAP1* gene region

Genomic coordinates (hg38)	Pearson R correlation with *BAP1* transcript levels	95% confidence interval	P-value
chr3:52,408,017	-0.79	-0.86 to -0.69	<0.0001
chr3:52,407,064	-0.46	-0.62 to -0.26	<0.0001
chr3:52,411,080	-0.45	-0.61 to -0.25	<0.0001
chr3:52,409,960	-0.27	-0.46 to -0.05	0.02
chr3:52,401,282	-0.27	-0.46 to -0.05	0.02
chr3:52,409,954	-0.27	-0.46 to -0.05	0.02
chr3:52,410,817	-0.24	-0.44 to -0.02	0.03
chr3:52,410,601	-0.10	-0.31 to 0.12	0.40
chr3:52,409,408	-0.08	-0.29 to 0.14	0.48
chr3:52,410,749	-0.07	-0.29 to 0.15	0.51
chr3:52,410,043	-0.07	-0.28 to 0.15	0.54
chr3:52,409,686	-0.07	-0.28 to 0.16	0.56
chr3:52,410,698	-0.06	-0.28 to 0.16	0.58
chr3:52,410,616	-0.06	-0.28 to 0.16	0.59
chr3:52,411,210	-0.06	-0.27 to 0.16	0.59
chr3:52,411,223	-0.01	-0.23 to 0.21	0.90
chr3:52,402,340	-0.01	-0.23 to 0.21	0.91
chr3:52,410,694	0.22	0.00 to 0.42	0.05
chr3:52,411,087	0.27	0.05 to 0.47	0.02

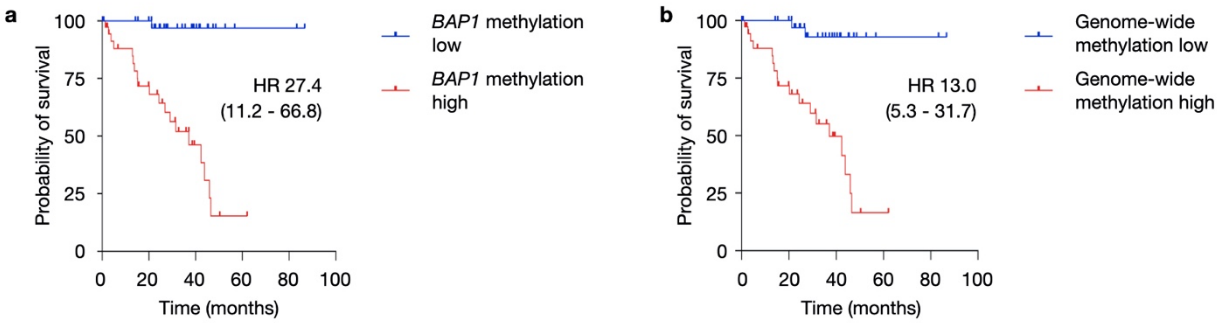

Supplementary figure 1. Global and *BAP1* methylation levels correlate with worse survival. A) Probability of survival of (n = 80) TCGA-UM subjects with primary tumors stratified by median *BAP1* β-methylation values or **B)** median β-methylation values obtained from the top 1% differentially methylated loci in monosomy 3 tumors. Statistical significance tested using two-sided log-rank test.
